# Parasternal electromyography in participants with mild or moderate chronic obstructive pulmonary disease in primary care: cohort study to assess technical and clinical application

**DOI:** 10.1136/bmjresp-2025-003418

**Published:** 2026-04-10

**Authors:** Timothy H Harries, Rebecca D’Cruz, Gill Gilworth, Chris Corrigan, Patrick Murphy, Nicholas Hart, Peter Schofield, Helen F Ashdown, Luke Daines, Patrick T White

**Affiliations:** 1 Department of Population Health Sciences, King’s College London, London, UK; 2 Lane Fox Respiratory Unit, Guy’s and St Thomas’ NHS Foundation Trust, London, UK; 3 Academic Department of Rehabilitation Medicine, University of Leeds, Leeds, UK; 4 Department of Asthma Allergy & Respiratory Science, King’s College London, London, UK; 5 Nuffield Department of Primary Care Health Sciences, University of Oxford, Oxford, UK; 6 Usher Institute, University of Edinburgh, Edinburgh, UK

**Keywords:** Pulmonary Disease, Chronic Obstructive, Perception of Asthma/Breathlessness, COPD epidemiology, Patient Outcome Assessment, Respiratory Measurement

## Abstract

**Background:**

Neural respiratory drive (NRD) measurement, reflecting the balance between respiratory muscle load and capacity, is quantified using surface parasternal electromyography (EMG_para_). EMG_para_ tracks recovery from severe exacerbations of chronic obstructive pulmonary disease (COPD). Among stable COPD participants, we hypothesised the existence of a relationship between NRD, breathlessness and airway obstruction. Study aims: (1) assessing the feasibility of measuring EMG_para_ in COPD participants with forced expiratory volume in 1 s (FEV_1_) ≥50% predicted in primary care; (2) investigating relationships between NRD measures, self-reported breathlessness, airflow obstruction severity and health-related quality of life (HRQoL).

**Methods:**

Participants with stable mild/moderate COPD, using inhaled corticosteroid (ICS) therapy, were recruited from 20 general practices. Participants were randomly allocated to continue using ICS (maintenance group) or to withdraw ICS (withdrawal group) over 6 weeks. EMG_para_, spirometry, self-reported breathlessness (modified Borg dyspnoea scale), COPD Assessment Test and Chronic Respiratory Disease Questionnaire Self-Administered Standardised were measured at baseline, 3- and 6-month follow-up. Bland-Altman plots examined agreement between serial measurements.

**Results:**

Forty COPD participants were recruited: age 70±9.2 years; body mass index 26±5.3 kg/m^2^; FEV_1_ 1.74±0.54 L; and FEV_1_% pred 69.6±14.0%. High-quality EMG_para_ data were obtained from 35 participants at baseline and 31 participants on three occasions. High intra-rater and inter-rater agreement for EMG_para_ (intraclass correlation coefficient >0.9) and moderate correlation between EMG_para_ and FEV_1_% predicted (r=−0.42; p=0.01) were recorded. No correlation was observed between resting EMG_para_ and breathlessness or HRQoL measures across the three time points.

**Conclusions:**

EMG_para_ measurement is feasible in primary care. In this group of COPD patients, lung function was stable across the three time points and EMG_para_ was associated with the degree of airflow obstruction. In the resting stable state in mild/moderate disease, there was no association between EMG_para_ and participant-reported outcomes. Further work should investigate the utility of EMG_para_ in mild/moderate COPD participants during acute exacerbation and recovery.

**WHAT IS ALREADY KNOWN ON THIS TOPIC**

- Non-invasive measurement of neural respiratory drive (parasternal electromyography (EMG_para_)) provides an objective, advanced physiological biomarker measure of the central control of breathing. It has been used extensively in research in secondary care to predict worsening and recovery from acute exacerbations of chronic obstructive pulmonary disease (COPD).

**WHAT THIS STUDY ADDS**

- This research describes the first use of EMG_para_ within primary care and in a group of over 30 stable COPD patients seen at consecutive visits over 6 months. Measurement of EMG_para_ is feasible in primary care, is stable and reproducible in this group of mild or moderate COPD patients.

**HOW THIS STUDY MIGHT AFFECT RESEARCH, PRACTICE OR POLICY**

- This study is an important step towards evaluating the utility of EMG_para_ measurement, reflecting change in neural respiratory drive, as a research tool to identify clinical deterioration in COPD patients in primary care.

## Background

 Chronic obstructive pulmonary disease (COPD) is a common respiratory condition that imposes substantial morbidity on participants and is the second most common cause of unscheduled healthcare attendance.^1^ The pathophysiological hallmark of COPD is expiratory airflow limitation, which arises as a consequence of airways inflammation, sputum and bronchoconstriction and drives static and dynamic hyperinflation.^2^ Hyperinflation leads to excess elastic, resistive and threshold loads on the respiratory muscle pump and reduces its capacity by impairing the force generation of the diaphragm. Neural respiratory drive (NRD), the central drive to breathe, increases in response to this imbalance.^3^

Neural output of the respiratory centres of the brainstem cannot be measured directly; therefore, surrogate measures are used to quantify NRD. Surface parasternal EMG (EMG_para_) has been used as a non-invasive advanced physiological biomarker to quantify NRD among stable COPD participants^3^ and during acute exacerbations of COPD (AECOPDs).^4^ EMG_para_ is a demonstrable, feasible measurement technique both in hospital and at home during severe exacerbations and recovery.^4 5^ Serial changes in EMG_para_ indices are associated with participant-reported and physician-defined clinical improvements or deteriorations during AECOPDs.^6 7^ Reproducibility of NRD and interobserver reliability have been demonstrated in COPD participants with severe or very severe airflow limitation in both the stable and exacerbating states.^8 9^ The European Respiratory Society has acknowledged that EMG_para_ shows promise as a non-invasive method to measure NRD.^10^ However, data are limited on the feasibility and reproducibility of EMG_para_ measurement among stable COPD participants with mild or moderate airflow obstruction in the primary care setting. The association between EMG_para_ and self-reported breathlessness and quality of life measures has not been reported within this stable group of COPD patients.

The aims of the study were to (1) assess the feasibility of measuring EMG_para_ in COPD participants with forced expiratory volume in 1 s (FEV_1_) ≥50% predicted in a primary care setting as part of a planned substudy of a randomised controlled feasibility trial^11^ and (2) investigate the relationships between NRD, self-reported breathlessness, degree of airway obstruction and quality of life.

## Methods

### Participant selection and exclusion criteria

This study was a nested observational cohort study within a feasibility randomised controlled trial of the withdrawal of inhaled corticosteroid (ICS) from Global Initiative for Chronic Obstructive Lung Disease (GOLD) Group A (mild) and GOLD Group B (moderate) COPD participants.^12^ The methods and results have been described previously.^11^ Inclusion criteria were: aged ≥45 years with a body mass index (BMI) <35 kg/m^2^; a documented history of COPD; evidence of mild or moderate post-bronchodilator airflow limitation (FEV_1_ ≥50% predicted and FEV_1_/forced vital capacity (FVC) ratio <0.7) within the previous 12 months and confirmed at recruitment; FEV_1_ reversibility ≤12% and ≤200 mL after bronchodilatation using 400 µg of salbutamol delivered via a spacer device^13^; having used ICS therapy (alone or in combination with a long-acting bronchodilator) daily for at least 3 months; fewer than two moderate exacerbations in the prior year; and no admissions to hospital with a severe exacerbation since the diagnosis of COPD was made. Eligible dosages of ICS therapy were defined as beclometasone dipropionate >400 µg/day or an equipotent dosage of another ICS.^14^ A moderate exacerbation of COPD was defined as treatment with antibiotics and/or oral corticosteroids in the community, and a severe exacerbation was defined by admission to hospital.^15^

Exclusion criteria include a current or medical history of asthma confirmed from the participant’s electronic record and at clinical interview; lung cancer or breathlessness secondary to cardiac disease; current severe mental illness (severe depression or psychosis) or dementia; current alcohol dependence; and continuous use of oral corticosteroids.

### Patient and public involvement

Patients and the public were first involved during the planning and application process of the feasibility trial within which this observational study was nested. A patient advisory group, drawn from the local British Lung Foundation Easybreath Group, was convened. This group provided advice to the project team on the writing of all patient literature and communication tools, on the development of patient data collection instruments, on the recruitment and retention of participants in the project and on the burden of the proposed investigations. A patient representative also joined the trial steering group. Members of the patient advisory group reviewed participant information sheets and informed the development of participant information resources.

### Data collection

#### Baseline assessment

Each participant’s medical history, current inhaler adherence (obtained through interview) and technique and lung function before and after bronchodilatation were recorded. Participants completed the COPD Assessment Test (CAT), Chronic Respiratory Disease Questionnaire Self-Administered Standardised (CRQ-SAS) and modified Borg (mBorg) dyspnoea scale. A CAT score of ≥10 points indicates high symptom burden^16^ and the minimum clinically important difference (MCID) is an increase in CAT score of ≥2, which indicates a deterioration in quality of life.^17^ The CRQ-SAS score consists of four domains: CRQ-dyspnoea domain, CRQ-fatigue domain, CRQ-emotional functioning domain, CRQ-mastery domain and the MCID is a change of −0.5 points per domain.^18^ The modified Borg scale for dyspnoea^19^ has an MCID ≥1 units.^18^ FEV_1_ and FVC were measured using a handheld spirometer (Micro, Carefusion, Basingstoke, UK) according to international standards.^20^ Each participant was randomly allocated to continue using ICS (maintenance group) or to gradually withdraw from ICS (withdrawal group) over 6 weeks.

### EMG_para_ measurement

EMG_para_ was measured as previously described.^4 5^ Each participant was in a seated position, with arms supported to minimise tonic activity of adjacent chest wall muscles. Following skin preparation, bipolar wet-gel electrodes (Ambu Blue Sensor Q; Ambu, St Ives, UK) were applied lateral to the sternum with one ground electrode on the lateral clavicle. Nasal cannulae positioned in subjects’ nostrils were connected to a differential pressure transducer (Pressure Sensor; TMSi) and identified the inspiration and expiration phases of respiration. EMG_para_ was recorded over 6 min of tidal breathing followed by maximal sniff manoeuvres for 1 min to measure maximal EMG_para_ (EMG_paramax_). Signals were amplified with a gain of 1000 and band-pass filtered at 1000–2000 Hz prior to acquisition and acquired using a 16-bit analogue-to-digital converter (Porti Physiological Amplifier; TMSi, Oldenzaal, the Netherlands).

Traces were analysed by converting raw EMG_para_ signals to root mean squared (RMS) using a moving window of 50 ms. Signals were analysed using Labchart software (AD Instruments, Chalgrove, UK). Digital traces were analysed by electronically stopping and holding the moving trace and reading with the naked eye the maximal EMG_para_ in each breath cycle. The peak RMS EMG_para_ activity for each inspiration was averaged over 6 min of tidal breathing and normalised to a value of sniff EMG_paramax_.^4^ Root mean square of the signal quantifies the intensity and duration of the muscle contraction and is linearly associated with increasing load on the muscles.^21^ Three NRD indices were derived: (1) mean EMG_para_; (2) EMG_para%max_, the mean peak inspiratory tidal EMG_para_ normalised to the maximal manoeuvre (division by the greatest EMG_para_ obtained during maximal sniff); and (3) NRDI (Neural Respiratory Drive Index), the product of EMG_para%max_ and respiratory rate.^4^ High-quality traces were continuous traces, with evidence of the maximal sniff and without interference from non-parasternal muscle activity.

### Follow-up visits

Follow-up visits took place at 3 and 6 months. The baseline measures, except for bronchodilator reversibility testing, were repeated at each assessment. Reports of disease exacerbations were verified by checking the electronic patient records.

### Statistical analysis

The sample size for the feasibility trial, in which this observational study was nested, was based on an estimation of the prevalence of patients who would deem it acceptable to be randomised to withdraw or continue their ICS therapy. We assumed that 90% of patients who had expressed interest in participating would find randomisation acceptable. At least 75 patients would have given 95% confidence of being within ±7% of the true figure of acceptability if the proportion who found randomisation acceptable was 90%. A power calculation was not undertaken for this observational study.

### Stability of EMG_para_ indices

Conformity of the continuous/parametric outcome measures with a parametric distribution was verified using the Shapiro-Wilk test. Parametrically distributed data are presented as the mean±SD. Continuous variables were assessed by analysis of variance with repeated measures. Bonferroni correction for multiple comparisons was made. The EMG_para_ indices were compared between visits and correlations were determined between baseline and 3 months visits, 3 months and 6 months visits, and baseline and 6 months visits. To determine the stability of the EMG_para_ indices across the assessments, Bland-Altman plots^22^ were generated. The correlation between variables at different visits was determined by using the Pearson correlation coefficient. Group comparisons of normally distributed data of the ICS withdrawal arm and usual care arm of the feasibility trial, at a single time point or between two time points, were analysed using the paired samples t-test. Categorical variables were compared using the χ^2^ test. Data were analysed using SPSS V.27.

### Reproducibility of NRD indices analysis

A random sample of 20 recordings was generated from 105 EMG_para_ traces. The sample included participants who had recordings at any time point across the three assessments (baseline, 3 months and 6 months) and one reading per participant was used. The analysis was carried out by two individuals (THH and RD). To determine intra-rater agreement, the same sample traces were analysed twice, after the interval of a week, by a single rater (THH). To determine inter-rater agreement, the second rater (RD) analysed each sample once. The first readings from rater one were compared with the readings from rater two. Intraclass correlation coefficients (ICC) using a two-way mixed effects model with absolute agreement tested were calculated for each of the variables and Bland-Altman plots^22^ were generated.

## Results

Forty participants were recruited (table 1). No bronchodilator inhalers had been used for 12 hours before the baseline assessment. EMG_para_ data were obtained from 35 participants at baseline. Missing baseline data were due to electrical interference in the recording of the parasternal muscle potential.

**Table 1:** Characteristics of participants at baseline

	Baseline n=40
Age (years): mean (SD)	70.1 (±9.2)
Male sex, n (%)	20 (50%)
BMI (kg/m^2^): mean (SD)	26.4 (±5.3)
Current smoker, n (%)	14 (35%)
Tobacco exposure (pack years): mean (SD)	33.5 (±20.8)
AECOPD in prior year: mean (SD)	0.5 (±0.5)
History of atopy, n (%)	26 (65%)
Currently on LABA+LAMA+ICS (%)	40 (100%)
Pre-bronchodilator FEV_1_ (L): mean (SD)	1.74 (±0.54)
Pre-bronchodilator FEV_1_ % predicted: mean (SD)	69.6 (±14.0)
Pre-bronchodilator FVC (L): mean (SD)	2.84 (±0.79)
Pre-bronchodilator FVC % predicted: mean (SD)	89.7 (±18.6)
Pre-bronchodilator FEV_1_/FVC: mean (SD)	0.61 (±0.09)
Post-bronchodilator FEV_1_ (L): mean (SD)	1.82 (±0.54)
Post-bronchodilator FEV_1_ % predicted: mean (SD)	72.9 (±13.6)
Post-bronchodilator FVC (L): mean (SD)	2.87 (±0.78)
Post-bronchodilator FVC % predicted: mean (SD)	90.8 (±18.8)
Post-bronchodilator FEV_1_/FVC: mean (SD)	0.63 (±0.07)
EMG_para_: mean (SD) (n=35)	5.9 (±2.1)
EMG_para%max_ (%): mean (SD) (n=35)	25.5 (±12.2)
NRDI (%.BPM): mean (SD) (n=35)	394.3 (±220.3)
CAT score: mean (SD)	16.3 (±7.8)
CRQ dyspnoea domain: mean (SD)	5.1 (±1.3)
CRQ fatigue domain: mean (SD)	4.0 (±1.5)
CRQ emotional functioning domain: mean (SD)	4.7 (±1.3)
CRQ mastery domain: mean (SD)	5.4 (±1.3)
Modified Borg score: mean (SD)	2.7 (±2.0)

AECOPD, acute moderate exacerbations of COPD; BMI, body mass index; CAT score, COPD Assessment Test; COPD, chronic obstructive pulmonary disease; CRQ dyspnoea, Chronic Respiratory Disease Questionnaire Self-Administered Standardised Dyspnoea score; EMG_para_, surface parasternal electromyography; FEV_1_, post-bronchodilatation forced expiratory volume in 1 s; FVC, post-bronchodilatation forced expiratory volume; ICS, inhaled corticosteroids; LABA, long-acting beta-agonist; LAMA, long-acting muscarinic antagonist; NRDI, Neural Respiratory Drive Index.

38 participants were followed up at 3 and 6 months (table 2). Three high-quality EMG_para_ readings (online supplemental figure S1) were obtained from 31 participants, two readings from five participants, one reading from two participants and no readings from two participants. One participant died during the study of an unrelated cause. One participant was lost to follow-up after baseline assessment. EMG_para_, NRDI, FEV_1_, FEV_1_% predicted, CAT, CRQ, mBorg scores did not differ between the three assessments (table 2). A decline of 8.7% (260 mL) was recorded in FVC over 6 months. There is no recognised MCID in FVC change for COPD.^18^ EMG_para%max_ was unchanged between baseline and 6 months, but decreased between 3 and 6 months. This was accompanied by a rise in maximal sniff, the denominator in the calculation of EMG_para%max_, between 3 and 6 months. Between baseline and 6 months there was no clinically significant change in symptom scores.

**Table 2:** Comparison of characteristics of participants at baseline, 3 and 6 months (analysis of variance with repeated measures)

	Baseline n=38	3 months n=38	6 months n=38	Baseline vs 3 months Difference between means (±95% CI)	3 vs 6 months Difference between means (±95% CI)	Baseline vs 6 months Difference between means (±95% CI)
Post-bronchodilator FEV_1_ (L): mean (SD)	1.90 (±0.56)	1.82 (±0.62)	1.81 (±0.54)	0.08 (−0.03 to 0.19) p=0.26	0.01 (−0.13 to 0.11) p=0.90	0.09 (−0.01 to 0.17) p=0.07
Post-bronchodilator FEV_1_ % predicted: mean (SD)	74.2 (±13.5)	72.1 (±17.6)	72.4 (±15.4)	2.1 (−1.9 to 6.2) p=0.59	−0.3 (−4.6 to 4.1) p=0.90	1.8 (−1.6 to 5.3) p=0.55
Post-bronchodilator FVC (L): mean (SD)	2.99 (±0.80)	2.81 (±0.75)	2.73 (±0.79)	0.18 (0.01 to 0.35) p=0.04	0.08 (−0.10 to 0.25) p=0.85	0.26 (0.11 to 0.39) p=0.001
Post-bronchodilator FVC % predicted: mean (SD)	92.1 (±19.3)	89.0 (±17.8)	86.8 (±18.1)	3.1 (−2.3 to 8.6) p=0.47	2.2 (−3.2 to 7.5) p=0.95	5.3 (0.6 to 9.9) p=0.02
Post-bronchodilator FEV_1_/FVC: mean (SD)	0.64 (±0.08)	0.65 (±0.12)	0.67 (±0.11)	−0.01 (−0.04 to 0.02) p=0.90	−0.02 (−0.05 to 0.006) p=0.16	−0.03 (0.002 to 0.06) p=0.04
EMG_para_ (µV): mean (SD) (n=31)	5.97 (±2.19)	5.81 (±2.38)	5.71 (±2.40)	0.16 (−0.56 to 0.88) p=0.78	0.10 (−0.58 to 0.87) p=0.71	0.26 (−0.70 to 1.21) p=0.67
EMG_para%max_ (%): mean (SD) (n=31)	25.4 (±12.9)	25.5 (±10.6)	22.0 (±10.4)	−1.1 (−4.9 to 4.7) p=0.96	3.5 (0.87 to 6.1) p=0.03	3.4 (−1.4 to 8.1) p=0.16
NRDI (%.BPM): mean (SD) (n=31)	394.7 (±232.2)	425.1 (±257.5)	377.5 (±213.7)	−30.4 (−159.2 to 98.5) p=0.55	47.6 (−56.8 to 152.1) p=0.77	17.2 (−68.9 to 103.5) p=0.62
Maximal sniff (µV): mean (SD)	28.5 (±14.6)	27.3 (±14.4)	32.0 (±17.8)	1.2 (−5.2 to 7.6) p=0.65	−4.7 (−8.9 to −0.4) p=0.03	−3.5 (−10.3 to 3.3) p=0.61
Respiratory rate (bpm)	15.79 (±4.64)	17.10 (±8.84)	17.18 (±4.49)	−1.31 (−4.60 to 1.98) p=0.96	−0.08 (−3.61 to 3.46) p=0.90	−1.39 (−2.80 to 0.02) p=0.06
CAT score: mean (SD)	15.6 (±7.3)	17.2 (±7.7)	16.8 (±8.0)	−1.6 (−5.0 to 1.8) p=0.71	0.4 (−2.8 to 4.2) p=0.58	−1.2 (−4.3 to 2.5) p=0.36
CRQ dyspnoea domain: mean (SD)	5.2 (±1.3)	5.3 (±1.4)	5.4 (±1.2)	−0.1 (−0.7 to 0.6) p=0.90	−0.1 (−0.8 to 0.5) p=0.49	−0.2 (−0.7 to 0.3) p=0.88
CRQ fatigue domain: mean (SD)	4.0 (±1.5)	3.9 (±1.5)	4.2 (±1.5)	0.1 (−0.4 to 0.7) p=0.90	−0.3 (−0.8 to 0.1) p=0.27	−0.2 (−0.6 to 0.2) p=0.84
CRQ emotional functioning domain: mean (SD)	4.7 (±1.3)	4.8 (±1.3)	5.1 (±1.4)	−0.1 (−0.6 to 0.4) p=0.81	−0.3 (−0.8 to 0.2) p=0.50	−0.4 (−0.8 to 0.1) p=0.20
CRQ mastery domain: mean (SD)	5.3 (±1.3)	5.0 (±1.5)	5.2 (±1.5)	0.3 (−0.3 to 1.0) p=0.71	−0.2 (−0.7 to 0.3) p=0.41	0.1 (−0.5 to 0.8) p=0.39
Modified Borg score: mean (SD)	2.6 (±1.8)	2.9 (±2.3)	2.8 (±1.9)	−0.3 (−1.4 to 0.8) p=0.47	0.1 (−1.1 to 1.3) p=0.84	−0.2 (−1.1 to 0.7) p=0.60

Adjustment for multiple comparisons: Bonferroni.

AECOPD, acute moderate exacerbations of COPD; BMI, body mass index; bpm, breaths per minute; CAT score, COPD Assessment Test; CRQ dyspnoea, Chronic Respiratory Disease Questionnaire Self-Administered Standardised Dyspnoea score; EMG_para_, surface parasternal EMG; EMG_para%max_, peak root mean squared EMG_para_ per inspiration divided by greatest EMG_para_ obtained during maximal sniff; FEV_1_, post-bronchodilatation forced expiratory volume in 1 s; FVC, post-bronchodilatation forced expiratory volume; ICS, inhaled corticosteroid; NRDI, Neural Respiratory Drive Index.

High levels of intra- and inter-rater reliability of the analysis of the tracings were detected, with an ICC >0.90 for each NRDI (tables3  4, online supplemental figures S2a–e and S3a–e).

**Table 3:** Intra-rater (one rater) agreement

Variable (n=20)	Test one mean (SD)	Test two mean (SD)	Difference (95% CI)*	ICC (95% CI)	SEM
EMG_para_ (µV)	5.14 (1.7)	5.18 (1.8)	0.04 (−0.21 to 0.13)	0.98 (0.95 to 0.99)	0.1
Maximal sniff	22.563 (9.9)	22.559 (9.9)	0.004 (−0.05 to 0.05)	1.00 (1.00 to 1.00)	0.02
Respiratory rate	15.67 (4.2)	15.82 (4.3)	0.15 (−0.33 to 0.02)	0.99 (0.98 to 0.99)	0.1
EMG_para%max_ (%)	26.85 (12.8)	26.96 (13.2)	0.11 (−1.06 to 0.83)	0.98 (0.97 to 0.99)	0.5
NRDI (%.BPM)	416.94 (239.1)	421.90 (249.8)	4.96 (−24.22 to 14.31)	0.98 (0.97 to 0.99)	9.2

* One-sample t-test.

EMG_para_, surface parasternal electromyography; EMG_para%max_, peak root mean squared EMG_para_ per inspiration/greatest EMG_para_ obtained at maximal sniff; ICC, intraclass correlation coefficient; NRDI, Neural Respiratory Drive Index.

**Table 4:** Inter-rater (two raters) agreement

Variable (n=20)	Test one mean (SD)	Test two mean (SD)	Difference (95% CI)*	ICC (95% CI)	SEM
EMG_para_ (µV)	5.15 (1.7)	5.23 (1.9)	0.08 (−0.37 to 0.21)	0.94 (0.86 to 0.98)	0.1
Maximal sniff	22.56 (9.9)	23.04 (10.6)	0.48 (−1.37 to 0.42)	0.98 (0.96 to 0.99)	0.4
Respiratory rate	15.67 (4.2)	15.91 (4.4)	0.24 (−0.56 to 0.07)	0.98 (0.97 to 0.99)	0.2
EMG_para%max_ (%)	26.85 (12.8)	27.06 (14.1)	0.21 (−1.78 to 1.36)	0.97 (0.92 to 0.99)	0.8
NRDI (%.BPM)	416.95 (239.1)	419.63 (251.3)	2.68 (−25.12 to 19.76)	0.98 (0.95 to 0.99)	10.7

* One-sample t-test.

EMG_para_, surface parasternal electromyography; EMG_para%max_, peak root mean squared EMG_para_ per inspiration/greatest EMG_para_ obtained at maximal sniff; ICC, intraclass correlation coefficient; NRDI, Neural Respiratory Drive Index.

The stability of the NRD measures (table 2) was assessed by generating Bland-Altman plots (online supplemental figures S4a–c, S5a,b and S6a–c). A Bland-Altman plot was not generated for EMG_para%max_ between 3 and 6 months, as the difference in values was significant.

There were no differences between the ICS withdrawal and ICS continuation groups at baseline or at 3 months or 6 months (online supplemental tables S1 and S2). There was no difference in the rate of moderate exacerbations between the ICS withdrawal and continuation groups. No severe exacerbations were recorded during the study. There was no evidence of variation in airflow or breathlessness across the three measurement points, either within the treatment groups or overall.

There were moderate correlations at the majority of time points between EMG_para_ and FEV_1_% predicted (r=−0.42; p=0.01), between EMG_para%max_ and FEV_1_% predicted (r=−0.46; p=0.005), and between NRDI and FEV_1_% predicted (r=−0.35; p=0.04) (table 5).

**Table 5:** Relationship between neural respiratory drive measures and FEV_1_% predicted at baseline, 3-, 6-month assessments (Pearson correlation co-efficient)

	FEV_1_% pred Baseline	FEV_1_% pred 3 months	FEV_1_% pred 6 months
EMG_para_(µV) Baseline	r=−0.28 p=0.11 n=35		
EMG_para_ (µV) 3 months		r=−0.42 p=0.01 n=34	
EMG_para_ (µV) 6 months			r=−0.42 p=0.01 n=36
EMG_para%max_ (%) Baseline	r=−0.37 p=0.03 n=35		
EMG_para%max_ (%) 3 months		r=−0.25 p=0.15 n=34	
EMG_para%max_ (%) 6 months			r=−0.46 p=0.005 n=36
NRDI (%.BPM) Baseline	r=−0.35 p=0.04 n=35		
NRDI (%.BPM) 3 months		r=−0.33 p=0.05 n=34	
NRDI (%.BPM) 6 months			r=−0.34 p=0.03 n=36

EMG_para_, surface parasternal electromyography; EMG_para%max_, peak root mean squared EMG_para_ per inspiration/greatest EMG_para_ obtained at maximal sniff; FEV_1_, forced expiratory volume in 1 s; NRDI, Neural Respiratory Drive Index.

No correlation was seen between EMG_para_ and any of the CAT (total score and breathlessness domain scores only), CRQ domains or mBorg scores. No correlation was seen between NRDI and CAT, CRQ domain scores. There were no consistent relationships between parasternal EMG indices and other subjective measures of breathlessness.

## Discussion

This study has demonstrated that it is feasible to conduct measurements of EMG_para_, to quantify NRD, in stable COPD participants with mild or moderate airflow obstruction in the primary care setting. The measurements had high inter- and intra-rater agreement, consistent with previous studies evaluating the application in secondary care of EMG_para_ in non-exacerbating COPD participants.^4 8 9^ EMG_para_, raw measures only, did not alter over time in COPD patients with stable airflow limitation and unchanged symptoms. Withdrawal of ICS therapy did not affect the measurements, as shown in online supplemental table S2.

COPD is characterised by expiratory airflow limitation,^23^ leading to increased end-expiratory lung volume and dynamic hyperinflation. Perturbations such as COPD exacerbations result in increased subjective work of breathing or breathlessness, anxiety and tachypnoea, and contribute to worsened dynamic hyperinflation.^24^ Change in EMG_para_ has been shown to correlate with change in mBorg breathlessness score during an AECOPD,^4^ to allow discrimination between those who clinically improved and those who deteriorated in hospital,^4^ and to assist in identifying risk of early hospital readmission following an AECOPD.^4 7^ These findings support the clinical usefulness of EMG_para_ measurement as a non-invasive advanced physiological marker in severe or very severe disease. In contrast, the population of the present study was assessed at rest and was in a stable state. We observed no correlation between EMG_para_ and measures of breathlessness and health-related quality of life (HRQoL) employed in participants with COPD. We observed a moderate correlation at the majority of time points between EMG_para_ and the degree of airways obstruction in the participants of the present study, as reflected by FEV_1_% predicted. This indicates the utility of EMG_para_ in evaluating the severity of load-capacity imbalance and NRD among stable COPD participants with mild or moderate airflow obstruction.

The EMG_para_ measures were stable over a 6-month period in this group of COPD patients whose FEV_1_ and reported symptom burden were unchanged during this time. This suggests that EMG_para_ is repeatable in this group of patients. To guide the application of this technology, future research should examine if there is a relationship between any changes in the EMG_para_ measures and changes in symptoms during periods such as exercise or clinical deterioration from a COPD exacerbation within this population. Breathlessness and its method of recording is highly subjective and may be affected by peripheral factors, including anxiety and depression,^25^ obesity,^26^ physical fitness^27^ and myasthenia gravis,^28^ among others. EMG_para_ may have a role as an objective measure of breathing among COPD participants with mild or moderate disease, with a baseline when the participant is well, against which worsening in breathing can be assessed, for example, during an acute exacerbation of the disease.^4 7^

The accurate diagnosis of an AECOPD is challenging due to the subjective definition of a COPD ‘exacerbation’ in contemporary international guidelines.^14^ It is possible that some of these moderate episodes would not be reflected by acutely elevated EMG_para_ signals. Future serial EMG_para_ measurement may identify a subset of COPD participants whose exacerbations truly reflect increasing airways obstruction and/or increased NRD. The use of serial EMG_para_ measurement may prove useful in these COPD participants in primary care. They may assist in identifying those at particular risk from a known COPD exacerbating factor, such as cigarette smoking. Other applications may include gauging the speed of progression of airways obstruction in individual participants objectively; correlating symptomatology with airways obstruction during episodes of ‘exacerbation’; assessing objectively the efficacy of interventions in participants with early, mild/moderate disease in terms of reducing EMG_para_ regardless of symptoms.

Strengths of the present study include that it was the first to investigate the use of EMG_para_ in primary care in a group of stable COPD participants with mild or moderate disease, followed up over 6 months. Limitations of the study include that these measurements were taken at rest. In a mild or moderate non-exacerbating COPD population, most symptoms will only occur on exertion. This may explain why a stronger association with airflow limitation and symptom scores was seen in other studies among COPD participants with higher severity disease,^4 7^ particularly where symptom scores report symptoms on exertion. Measures of airflow and lung volume were not undertaken while recording EMG_para_. Variation in EMG_para_ between visits may have been due, in part, to differences in lung volume and flow rates at different time points. Two raters determined the inter-rater agreement. Serial EMG_para_ measures in primary care can be assessed. Currently, data analysis relies on expert visual analysis of the generated traces. The raw EMG_para_ signal depends on the size, type and depth of muscle as well as on the quality of the signal generated.^24^ The quality of the EMG_para_ signal generated is dependent on the quality of the electrode contact with the skin, the positioning of the sternal electrodes and interference from other physiological electrical activity, such as voluntary and involuntary activity from adjacent muscles.^24^

An automated algorithm which could identify the peak inspiratory tidal EMG_para_ would simplify and standardise the analysis, increasing the practicality of its use and the likelihood of its implementation in primary care. Though the findings are consistent with prior research,^8 9^ they require confirmation within a larger study.

## Conclusions

The measurement of EMG_para_ is feasible and reproducible in stable COPD participants with mild or moderate airflow obstruction in the primary care setting. The next step is to assess its accuracy in detecting a change in breathlessness in similar patients by measurement during acute exacerbation or on exertion. This will be essential in predicting its future role in the assessment of the impact of treatment in trials that include COPD participants with mild or moderate disease.

## Supplementary material

10.1136/bmjresp-2025-003418 online supplemental file 1

## Data Availability

Data are available upon reasonable request.
