# Supplementary material for: Parasternal electromyography in participants with mild or moderate chronic obstructive pulmonary disease in primary care: cohort study to assess technical and clinical application
Source: BMJ Open Respir Res. 2026 Apr 10;13(1):e003418. doi: 10.1136/bmjresp-2025-003418 (PMC13084930; doi:10.1136/bmjresp-2025-003418)
Supplement: online supplemental file 1 [file bmjresp-13-1-s001.docx]

**Parasternal electromyography in participants with mild or moderate chronic obstructive pulmonary disease in primary care: technical and clinical application**

Timothy H Harries*^1^, Rebecca F D’Cruz^2^, Gill Gilworth^3^, Christopher J Corrigan^4^, Patrick B Murphy^2^, Nicholas Hart^2^, Peter Schofield^1^, Helen Ashdown^5^, Luke Daines^6^, Patrick T White^1^

**Supplementary File**


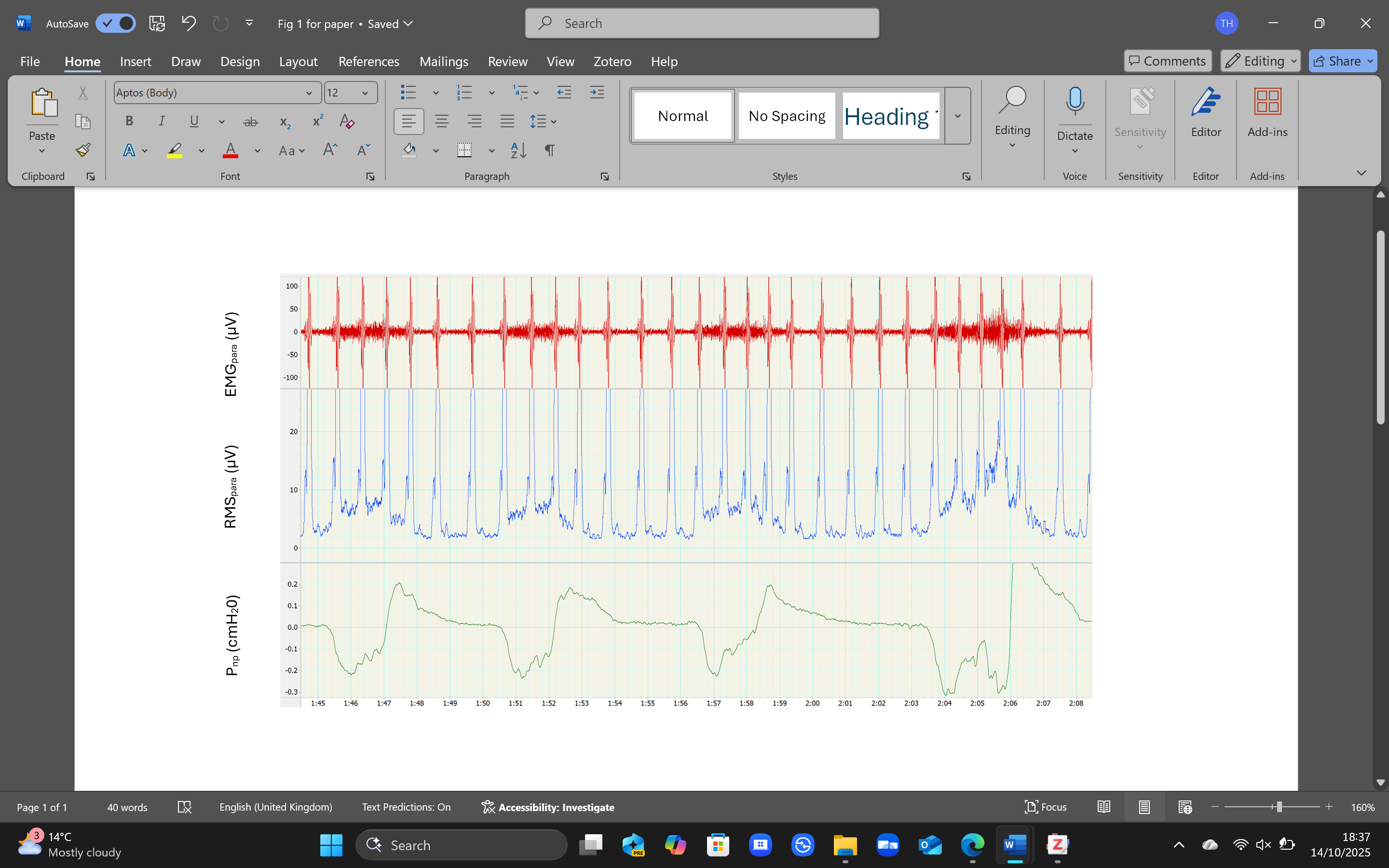


**Figure S1: Representative trace of raw data of nasal pressure and second intercostal space parasternal electromyogram during tidal breathing in a patient with stable chronic obstructive pulmonary disease. P_np_, nasal pressure derived from nasal cannulae; EMG_para,_ parasternal electromyogram activity; RMS_para_, root mean squared analysis of EMG_para._**


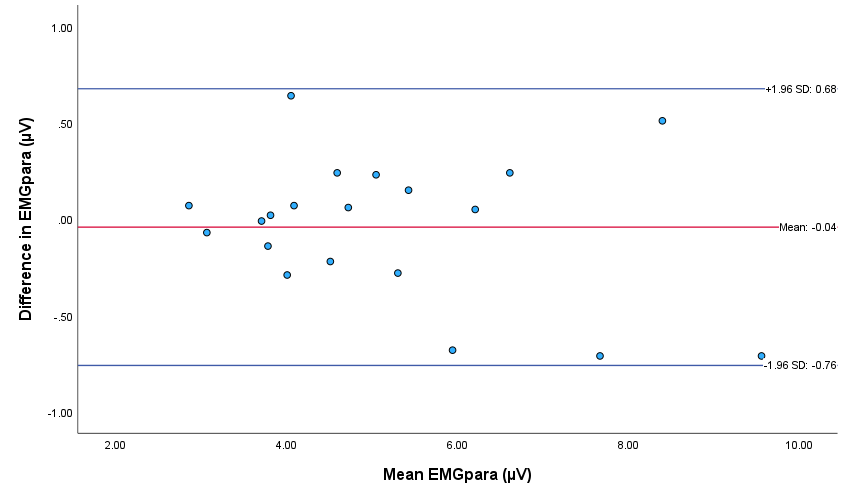


**Figure S2a Bland-Altman plot of intra-rater agreement (EMG_para_)**


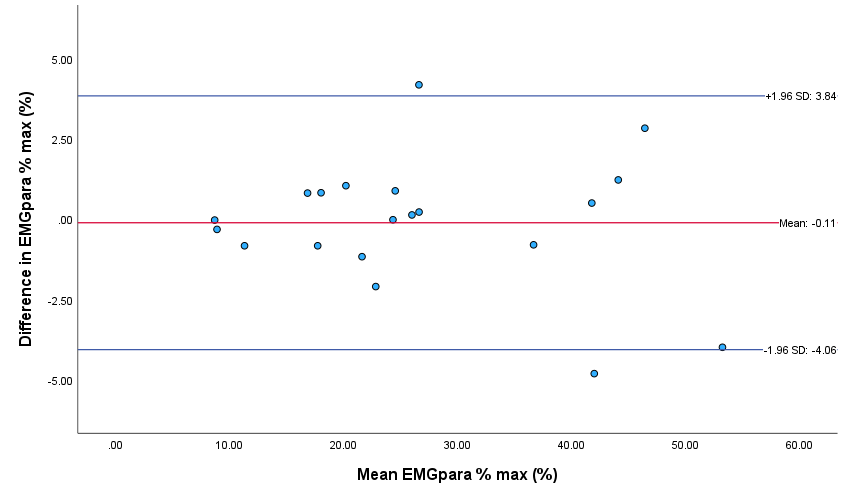


**Figure S2b Bland-Altman plot of intra-rater agreement (EMG_para%max_)**


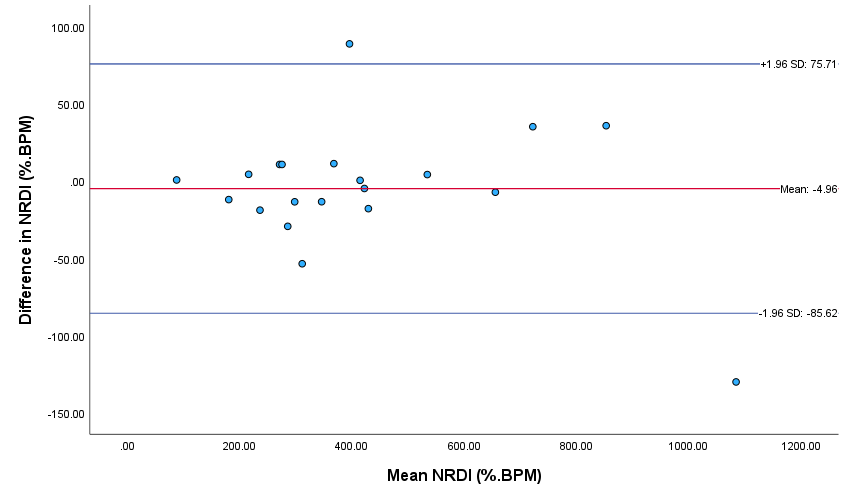


**Figure S2c Bland-Altman plot of intra-rater agreement (NRDI)**


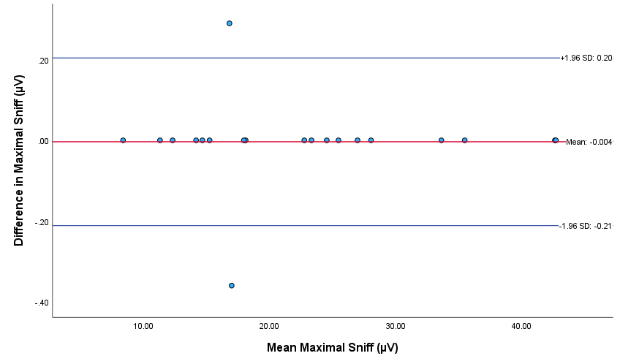


**Figure S2d Bland-Altman plot of intra-rater agreement (Maximal sniff)**


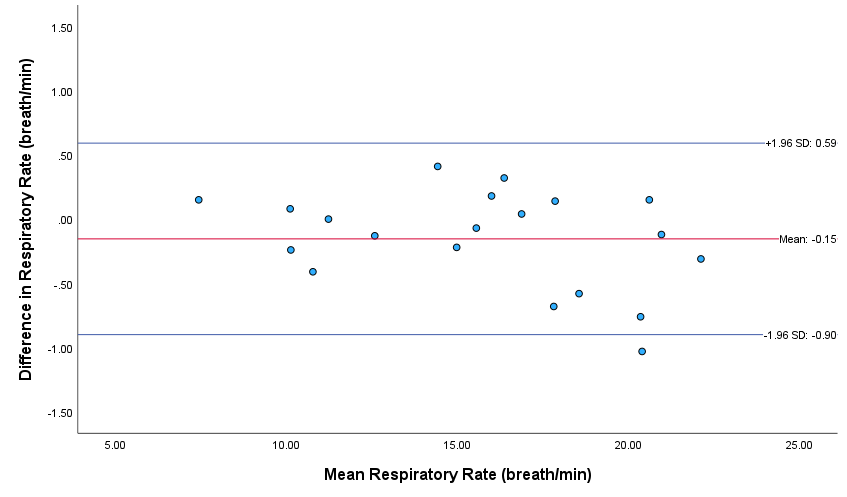


**Figure S2e Bland-Altman plot of intra-rater agreement (Respiratory rate)**


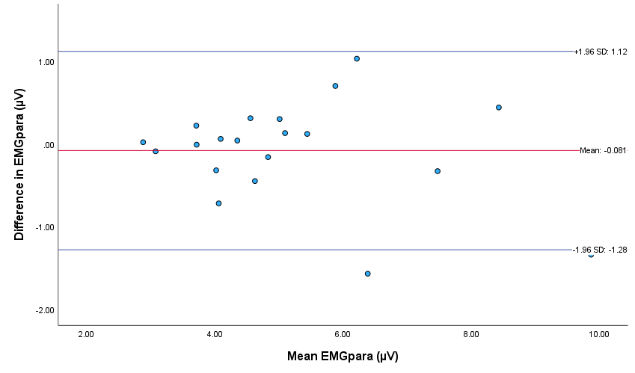


**Figure S3a Bland-Altman plot of inter-rater agreement (EMG_para_)**


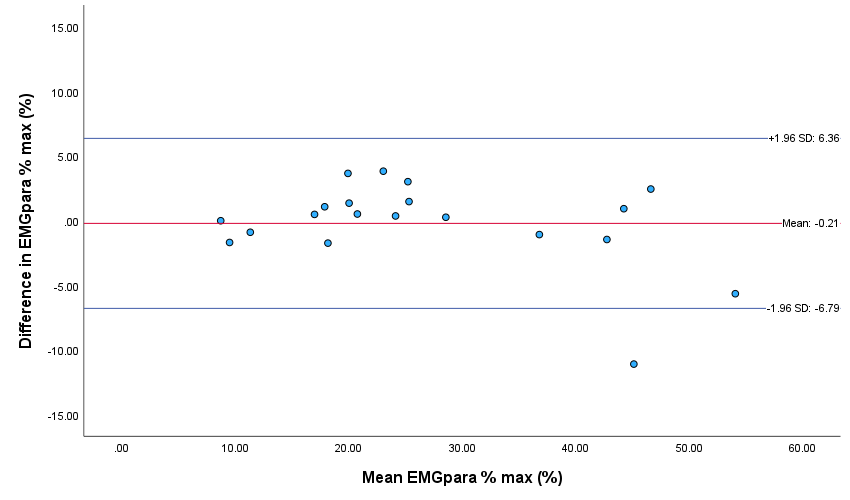


**Figure S3b Bland-Altman plot of inter-rater agreement (EMG_para%max_)**


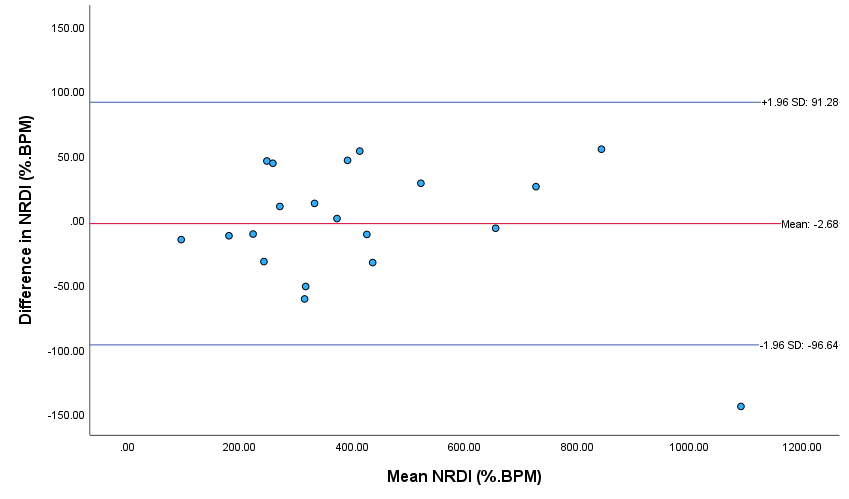


**Figure S3c Bland-Altman plot of inter-rater agreement (NRDI)**


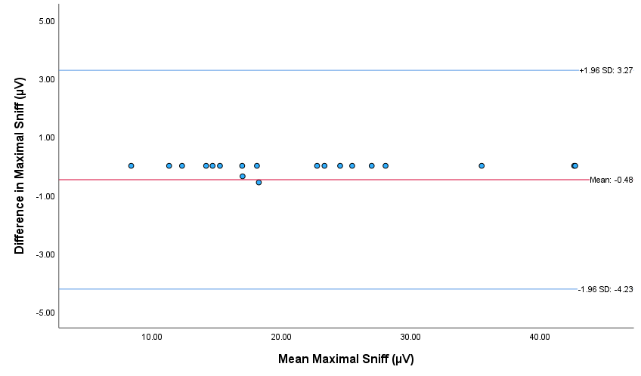


**Figure S3d Bland-Altman plot of inter-rater agreement (Maximal sniff)**


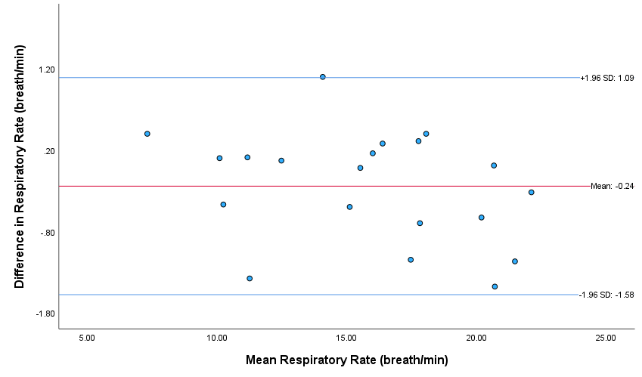


**Figure S3e Bland-Altman plot of inter-rater agreement (Respiratory rate)**


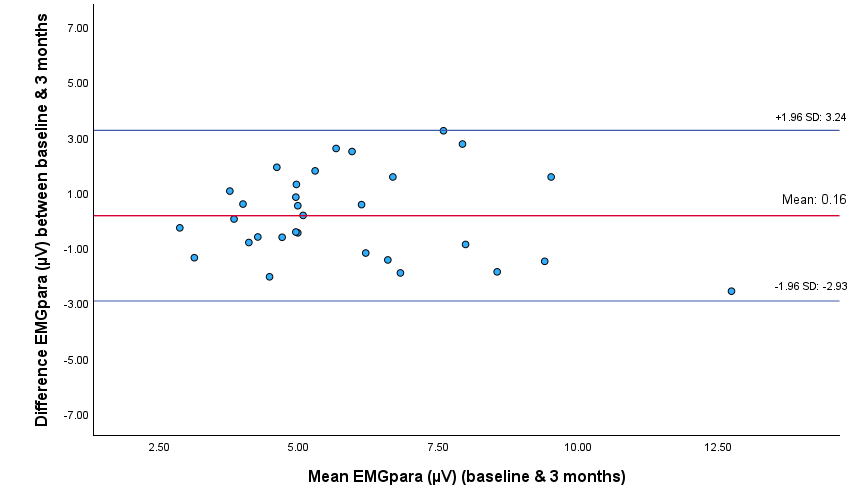


**Figure S4a: Bland-Altman plot of agreement between EMG_para_ readings (baseline & 3 months)**


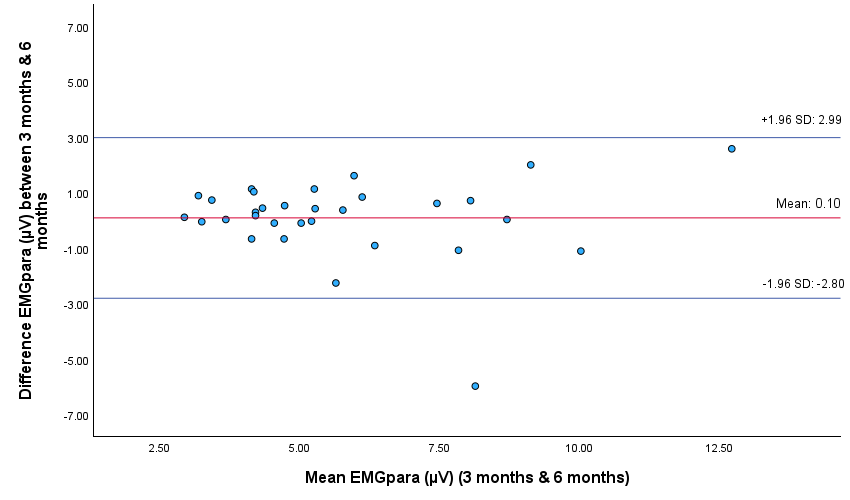


**Figure S4b: Bland-Altman plot of agreement between EMG_para_ readings (3 months & 6 months)**


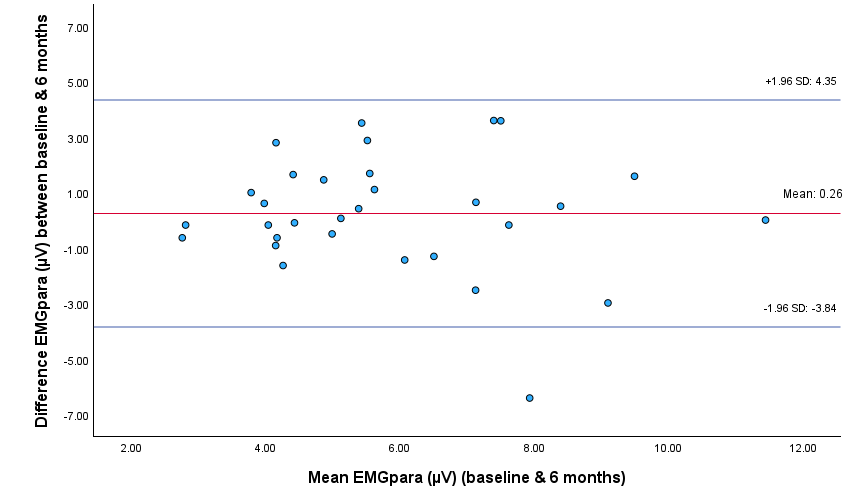


**Figure S4c: Bland-Altman plot of agreement between EMG_para_ readings (baseline & 6 months)**


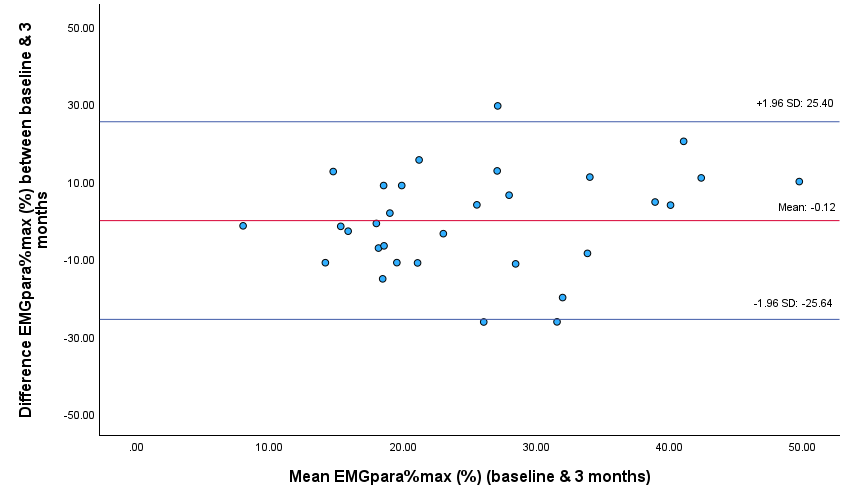


**Figure S5a: Bland-Altman plot of agreement between EMG_para%max_ readings (baseline & 3 months)**


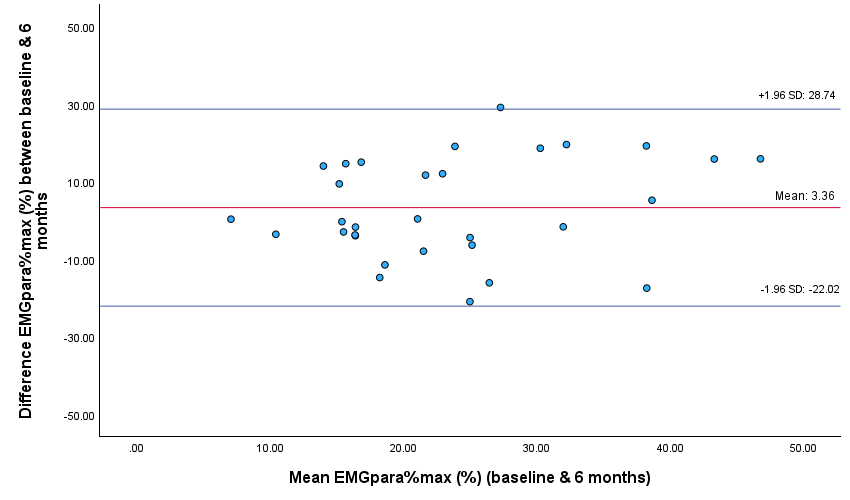


**Figure S5b: Bland-Altman plot of agreement between EMG_para%max_ readings (baseline & 6 months)**


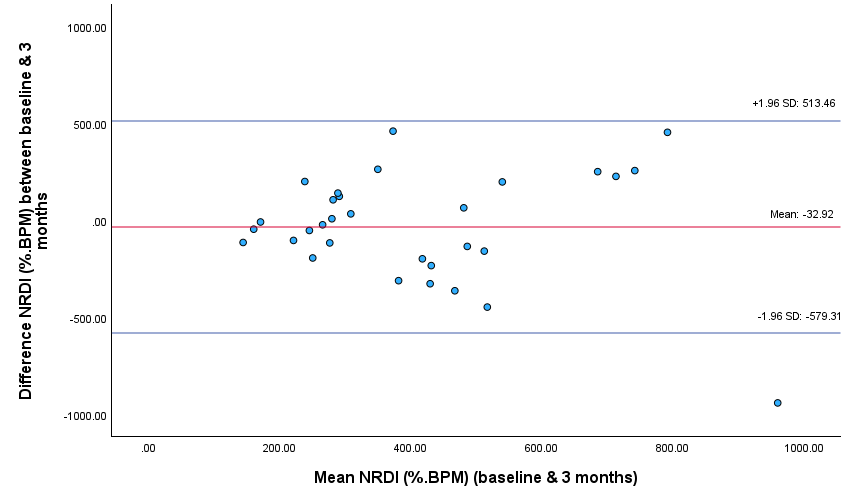


**Figure S6a: Bland-Altman plot of agreement between NRDI readings (baseline & 3 months)**


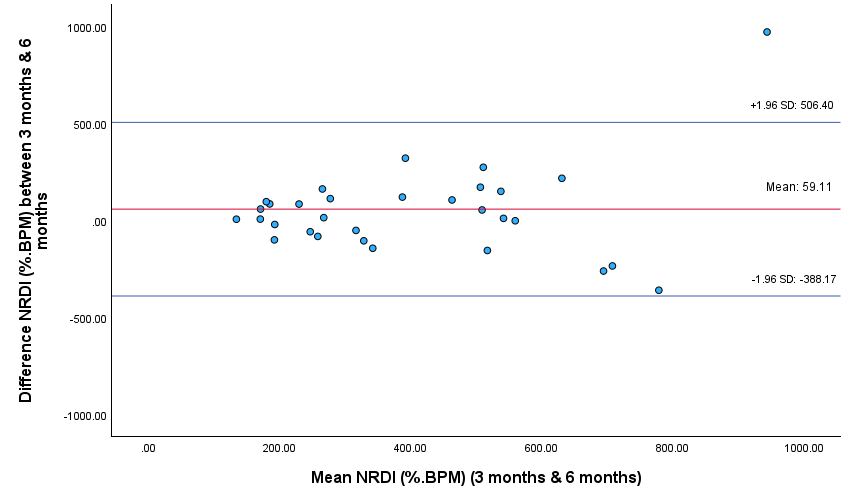


**Figure S6b: Bland-Altman plot of agreement between NRDI readings (3 months & 6 months)**


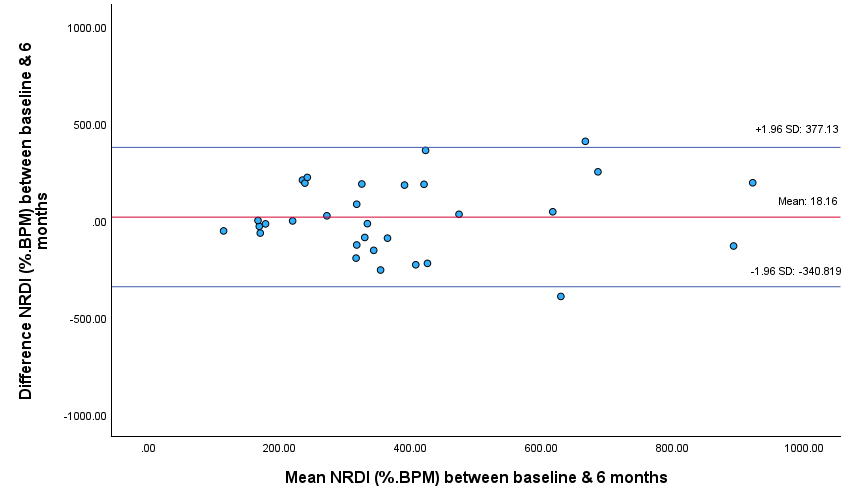


**Figure S6c: Bland-Altman plot of agreement between NRDI readings (baseline & 6 months)**

**TABLE S1. Characteristics at baseline of all participants, participants allocated to withdrawal from ICS^✣^ and those allocated to usual care, and outcome of comparisons (paired t-test or Chi-squared test) between withdrawal and usual care groups**

|  | **All participants**  **n=40** | **ICS withdrawal^✣^**  **n=20** | **Usual care**  **n=20** | **p-value** |
| --- | --- | --- | --- | --- |
| **Age (years): mean (SD)*** | 70.10 (±9.22) | 71.07 (±8.33) | 69.19 (±10.16) | 0.53 |
| **Male sex, n (%)†** | 20 (50%) | 10 (50%) | 10 (50%) | 1.00 |
| **BMI (kg/m^2^): mean (SD)*** | 26.40 (±5.29) | 26.09 (±5.55) | 26.72 (±5.15) | 0.71 |
| **Current smoker, n (%)†** | 14 (35%) | 6 (30%) | 8 (40%) | 0.44 |
| **Tobacco Exposure (Pack Years): mean (SD)*** | 33.47 (±20.79) | 29.83 (±17.96) | 37.11 (±23.20) | 0.29 |
| **AECOPD in prior year: mean (SD)*** | 0.48 (±0.51) | 0.50 (±0.51) | 0.45 (±0.51) | 0.76 |
| **History of Atopy, n (%)†** | 65% | 75% | 55% | 0.18 |
| **Pre-bronchodilator**  **FEV_1_ (L): mean (SD)*** | 1.74 (±0.54) | 1.68 (±0.46) | 1.80 (±0.49) | 0.52 |
| **Post-bronchodilator**  **FEV_1_ (L): mean (SD)*** | 1.82 (±0.54) | 1.77 (±0.47) | 1.87 (±0.61) | 0.58 |
| **Pre-bronchodilator**  **FEV_1_ % predicted: mean (SD)*** | 69.63 (±14.03) | 69.20 (±14.41) | 70.05 (±13.99) | 0.85 |
| **Post-bronchodilator**  **FEV_1_ % predicted: mean (SD)*** | 72.85 (±13.56) | 73.53 (±14.12) | 72.79 (±13.70) | 0.98 |
| **Pre-bronchodilator**  **FVC (L): mean (SD)*** | 2.84 (±0.79) | 2.68 (±0.67) | 3.00 (±0.88) | 0.19 |
| **Post-bronchodilator**  **FVC (L): mean (SD)*** | 2.87 (±0.78) | 2.72 (±0.68) | 3.03 (±0.87) | 0.23 |
| **Pre-bronchodilator**  **FEV_1_/FVC: mean (SD)*** | 0.61 (±0.09) | 0.63 (±0.09) | 0.60 (±0.10) | 0.36 |
| **Post-bronchodilator**  **FEV_1_/FVC: mean (SD)*** | 0.63 (±0.07) | 0.65 (±0.05) | 0.62 (±0.08) | 0.23 |
| **Currently on LABA+LAMA+ICS (%)†** | 100% | 100% | 100% | 1.00 |
| **EMG_para_: mean (SD)* (n=35)** | 5.9 (±2.10) | 6.1 (±2.07) | 5.77 (±2.18) | 0.68 |
| **EMG_para_ % MAX: mean (SD)* (n=35)** | 25.49 (±12.20) | 27.69 (±14.50) | 23.41 (±10.00) | 0.31 |
| **NRDI: mean (SD)* (n=35)** | 394.34 (±220.34) | 450.12 (±252.25) | 341.66 (±176.52) | 0.15 |
| **CAT score: mean (SD)*** | 15.76 (±7.54) | 17.84 (±6.87) | 13.68 (±7.79) | 0.08 |
| **CRQ dyspnoea domain: mean (SD)*** | 5.1 (±1.3) | 5.16 (±1.30) | 5.1 (±1.4) | 0.87 |
| **CRQ fatigue domain: mean (SD)*** | 4.11 (±1.42) | 4.04 (±1.15) | 4.18 (±1.69) | 0.67 |
| **CRQ emotional functioning domain: mean (SD)*** | 4.80 (±1.25) | 4.81 (±1.06) | 4.79 (±1.44) | 0.93 |
| **CRQ mastery domain: mean (SD)*** | 5.4 (±1.3) | 5.1 (±1.4) | 5.7 (±1.1) | 0.10 |
| **Modified Borg score: mean (SD)*** | 2.7 (±2.0) | 3.4 (±1.9) | 2.1 (±1.8) | 0.03 |
| **^✣^**inhaled corticosteroids; *t-test; †Chi-squared test. SD: standard deviation. BMI: Body Mass Index. CAT score: COPD Assessment Test. FEV_1_: Post-bronchodilatation Forced Expiratory Volume in 1 second. FVC: Post-bronchodilatation Forced Expiratory Volume. AECOPD: acute moderate exacerbations of COPD. CRQ dyspnoea: Chronic Respiratory Disease Questionnaire Self-Administered Standardized Dyspnoea score. LABA: long-acting beta-agonist. LAMA: long-acting muscarinic antagonist. ICS: inhaled corticosteroid. AECOPD: moderate exacerbation of COPD (max of 1 for inclusion in trial). | | | | |

**Table S2. Comparison of withdrawal and usual care groups at 3 and 6 months: lung function, quality of life and breathlessness (paired t-test)**

| **Measure** | **3 months** | | | **6 months** | | |
| --- | --- | --- | --- | --- | --- | --- |
|  | **Withdrawal**  **n=19** | **Usual care**  **n=19** | **p-value** | **Withdrawal**  **n=19** | **Usual care**  **n=19** | **p-value** |
| **FEV_1_ % predicted: mean (SD)*** | 70.95 (±18.22) | 73.94 (±15.13) | 0.59 | 72.00 (±16.59) | 71.63 (±12.63) | 0.94 |
| **FEV_1_/FVC: mean (SD)*** | 0.66 (±0.09) | 0.64 (±0.13) | 0.72 | 0.68 (±0.11) | 0.66 (±0.12) | 0.49 |
| **AECOPD during trial: mean (SD)*** | 0.20 (±0.41) | 0.10 (±0.31) | 0.39 | 0.15 (±0.37) | 0.05 (±0.22) | 0.30 |
| **EMG_para_: mean (SD)* (n=31)** | 5.59 (±1.65) | 6.00 (±2.87) | 0.61 | 5.45 (±2.13) | 6.07 (±2.58) | 0.44 |
| **EMG_para_ % MAX: mean (SD)* (n=31)** | 25.78 (±12.35) | 28.03 (±10.06) | 0.57 | 22.12 (±12.21) | 23.15 (±6.91) | 0.76 |
| **NRDI: mean (SD)* (n=31)** | 401.52  (±184.39) | 503.46  (±321.71) | 0.26 | 396.70  (±252.02) | 372.75  (±139.15) | 0.73 |
| **CAT score: mean (SD)*** | 18.90 (±7.56) | 15.24 (±7.64) | 0.16 | 17.16 (±6.78) | 16.42 (±9.28) | 0.78 |
| **CRQ dyspnoea domain: mean (SD)*** | 5.1 (±1.2) | 5.5 (±1.6) | 0.36 | 5.4 (±1.1) | 5.6 (±1.3) | 0.62 |
| **CRQ fatigue domain: mean (SD)*** | 3.67 (±1.32) | 4.18 (±1.69) | 0.32 | 4.29 (±1.28) | 4.44 (±1.65) | 0.76 |
| **CRQ emotional functioning domain: mean (SD)*** | 4.81 (±1.21) | 4.84 (±1.43) | 0.94 | 5.41 (±1.04) | 4.81 (±1.67) | 0.20 |
| **CRQ mastery domain: mean (SD)*** | 4.7 (±1.3) | 5.5 (±1.4) | 0.10 | 5.2 (±1.2) | 5.4 (±1.6) | 0.70 |
| **Modified Borg score: mean (SD)*** | 3.1 (±2.1) | 2.4 (±2.5) | 0.39 | 3.1 (±1.7) | 2.4 (±2.1) | 0.28 |
| *t-test. FEV_1_: forced expiratory volume in first second; SD: standard deviation; CAT: COPD assessment test; AECOPD: acute moderate exacerbations of COPD. CRQ-SAS: Chronic Respiratory Disease Questionnaire Self-Administered Standardized Dyspnoea, Fatigue, Emotional functioning, Mastery scores. | | | | | | |
